# *Citrus aurantium* ‘Changshan-huyou’—An ethnopharmacological and phytochemical review

**DOI:** 10.3389/fphar.2022.983470

**Published:** 2022-09-02

**Authors:** Liang Gao, Hui Zhang, Chun-Hui Yuan, Ling-Hui Zeng, Zheng Xiang, Jian-Feng Song, Hua-Gang Wang, Jian-Ping Jiang

**Affiliations:** ^1^ School of Medicine, Zhejiang University City College, Hangzhou, China; ^2^ College of Pharmaceutical Science, Zhejiang University of Technology, Hangzhou, China; ^3^ Quzhou Institute for Food and Drug Control, Quzhou, China; ^4^ Zhejiang Jing Yuetang Pharmaceutical Co. LTD, Shaoxing, China

**Keywords:** *Citrus aurantium* ‘Changshan-huyou’, chemical constituents, pharmacological activities, flavonoids, antioxidant, anti-inflammatory

## Abstract

Citrus fruits are composed of oil cells layer, white membrane layer, pulp and seeds. The cultivar *Citrus aurantium* ‘Changshan-huyou’ (CACH) is a hybridization of *Citrus grandis* Osbeck and *C. sinensis* Osbeck. It is a rutaceae plant, and mainly grows in Changshan, Zhejiang, China. With the exploration of its high traditional values, it has been paid more and more attention by the scientific community in recent years. At present, one hundred and two chemical constituents have been identified from the pulp and peel of CACH, including volatile oils, terpenoids, phenols, limonins, sugars, etc., As the representative active component of CACH, phenols have been widely investigated. Studies have shown that CACH shows a variety of significant pharmacological activities, such as anti-inflammatory, antioxidant, hepatoprotective activity, respiratory system protection and intestinal regulation activity. This review mainly introduces the chemical constituents and pharmacological activities of CACH, and discusses its future research and development directions. It will provide theoretical basis for further research of its pharmacodynamic substances, functional mechanism and rational utilization.

## 1 Introduction

**FIGURE 1:**
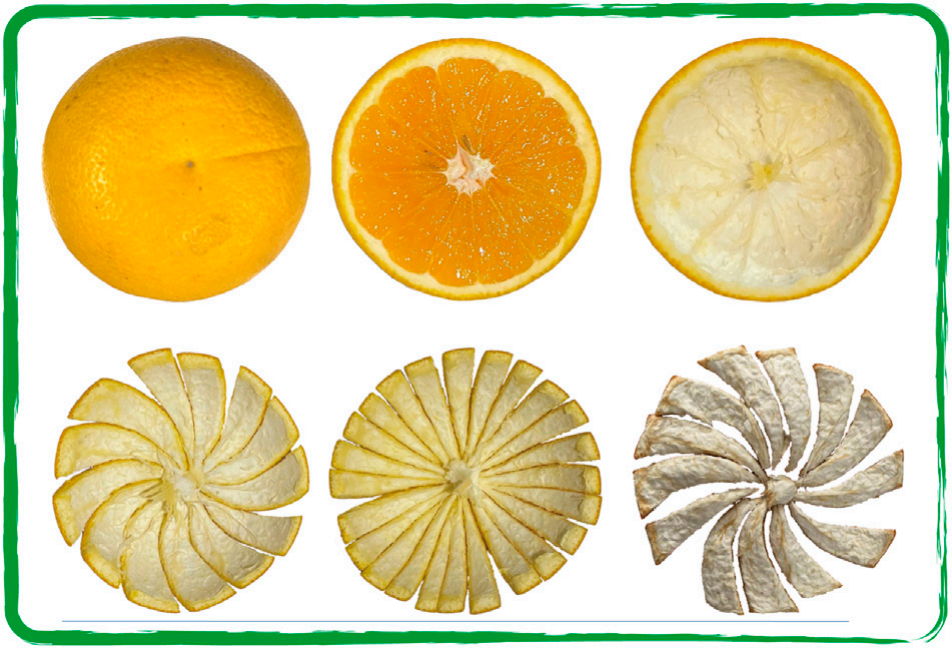
Surface and internal structure of CACH.

Citrus fruits have a unique structure among fruits, it is composed of oil cells layer, white membrane layer, pulp and seeds (Ranganna et al., 1983). They are particularly good sources of vitamin C, folic acid and important bioactive polyphenols. These substances maintain the integrity of the body’s immune barrier and inhibit inflammation in multiple ways (Miles and Calder, 2021). The cultivar *Citrus aurantium* ‘Changshan-huyou’ (CACH) is a kind of folk plant (Figure 1). It is a hybridization of *Citrus grandis* Osbeck and *C. sinensis* Osbeck, and mainly grows in Changshan, Quzhou, Kecheng, Longyou of Zhejiang province. It has been cultivated for hundreds of years in China. It grows rapidly, and its fruit expands mainly in June to July and September. Its expansion rate slows down after mid-September and harvested in November (Hu et al., 2020). It has the characteristics of high yield, frost resistance, strong adaptability and storage resistance.

Qu Zhi Qiao (QZQ), the immature dry fruit of CACH, is a good source of flavonoids. Modern pharmacological studies had shown that QZQ also had effects of antioxidant (Sun et al., 2005; Xu et al., 2010; Fang et al., 2018; Shi et al., 2020), anti-inflammatory (Jiang et al., 2019; Hu et al., 2020), anti-tumor (Bellocco et al., 2009), hypoglycemic (Zhang et al., 2012), protection on organs (Hwang and Yen, 2008; Vazhappilly et al., 2019), etc., It is a variety included in “The Processing Standards of Traditional Chinese Medicine of Zhejiang Province” (2015 Edition). In addition, it was listed as the new “Zhejiang Eight Flavors” and“Quzhou six Flavors”cultivars cultivation catalog of Zhejiang province in 2018. In 2019, it was selected into Chinese Agricultural Brand Catalogue, and was selected into the second batch of China-Europe Geographical Indications Protection List in the next year. All these mean that it has become the key development and support variety of medicinal materials in Zhejiang province.

**FIGURE 2:**
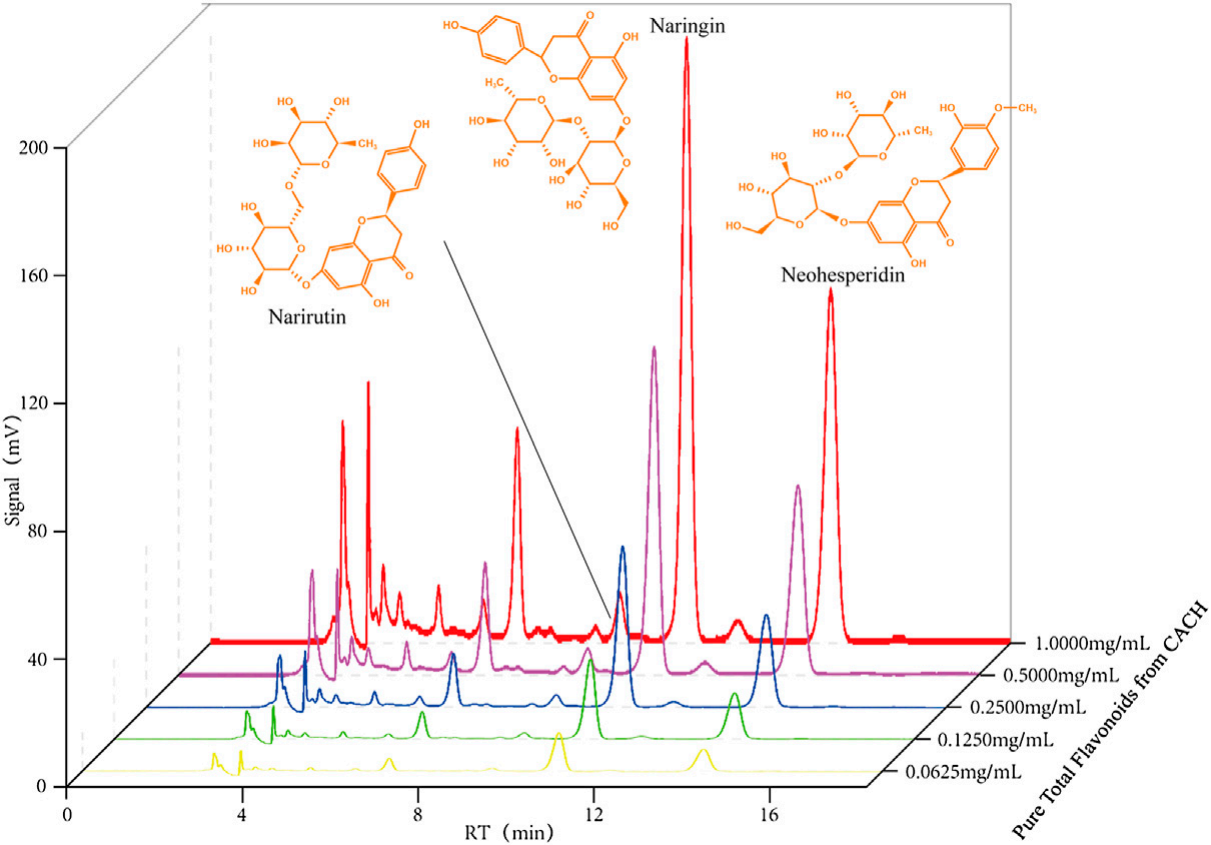
Main flavonoids isolated from CACH.

With the development of modern scientific research, the scientific community has a deeper understanding of the biological activities of citrus fruits, which makes them more popular in the world (Food And Agriculture Organization, 2017). CACH is cheap and produces huge quantities, it has great potential for research and development. The project team established the preparation method of pure total flavonoids from citrus (PTFC) in the early stage. It was confirmed that its flavonoid chemicals mainly included naringin, neohesperidin, narirutin, etc., (Jiang et al., 2014) (Figure 2). With the increasing yield of CACH in recent years, the sideline products are often treated as waste. It increases the production and operation cost and presents a negative impact on the environment (Singh et al., 2020). Studies have confirmed that CACH shows a variety of biological activities, but there is no clear summary of traditional usage, chemical components and pharmacological effects. Therefore, the paper reviews the research progress of them at home and abroad, aiming to provide guidance for further research and reference for its product development and comprehensive utilization.

## 2 Materials and methods

The information about the traditional usages, phytochemicals and pharmacological properties of CACH was searched via PubMed, Web of Science, Google Scholar, China National Knowledge Infrastructure (CNKI) and Springer search using Chinese or English as the retrieval languages. The keywords used include Changshan-huyou, Qu Zhi Qiao/Ke, traditional usages, phytochemistry, chemical constituents, pharmacological effects and other related words. All references of the review were from experimental studies and published prior to May 2022 were reviewed. All figures of the review are uploaded in TIF format with 300 dpi resolution and RGB color mode. All chemical structures of the review were drawn using ChemDraw 18.0 software.

## 3 Traditional usage

CACH, a plant of rutaceae, was first recorded in Qu Zhou Fu Zhi (Qing dynasty, A.D. 1711). The ancient book clearly records that Changshan is the birthplace of CACH, its history could go back to over six hundred years ago. Jiang Shan Xian Zhi (Qing Dynasty, A. D. 1873) and Chang Shan Xian Zhi (Qing Dynasty, A. D. 1886) also classified the local citrus varieties, these records confirmed the long cultivation history of CACH. It has been used as medicine in Changshan for more than 100 years. Its pulp, peel and processed products traditionally and ethnically used as medicine for a long time, and it has also been recorded in Xin Xiu Ben Cao (Tang Dynasty, He, 2013), Ben Cao Qiu Yuan (Qing dynasty, Zhao, 2016), Ben Cao Gang Mu (Ming dynasty, Wang, 2005). From the perspective of traditional Chinese medicinal (TCM), CACH tastes spicy and bitter, and has the functions of clearing away heat and promoting blood circulation. The channel tropisms are spleen and stomach meridians. Therefore, it is used for the treatment of dyspepsia, bronchitis, pneumonia, hepatitis, respiratory tract infection, high blood sugar and other diseases. The processing of QZQ follows the ancient process of fructus aurantii. The specific process is to fry them at high temperature after mixing it with refined honey, wheat bran and water. There is a tradition of boiling the pulp of CACH and rock sugar with water in Changshan, which can prevent and treat cough (Zhe, 2016). There are also some other folk remedies. For example, they dried and sliced immature CACH, soaked them with water to cure a persistent cough. In addition, QZQ has been used for a long time in the folk treatment of children’s respiratory tract infection in the local, and its efficacy is clear. Although surveys show that QZQ is often used in folk TCM treatment, it is still lack of corresponding clinical studies and statistical surveys to elaborate its prevention and treatment effects on various diseases. However, it is exciting that a recent invention (CN202110880713.1, You et al., 2021) with QZQ as the main ingredient for the treatment of viral pneumonia is under review. This invention proposed the preparation and application of its active ingredient. More importantly, it means that people are paying more attention to QZQ. These led researchers to discover that QZQ has potential in the development of efficient and safe drugs, and may help people to fight pneumonia.

## 4 Chemical constituents

**FIGURE 3:**
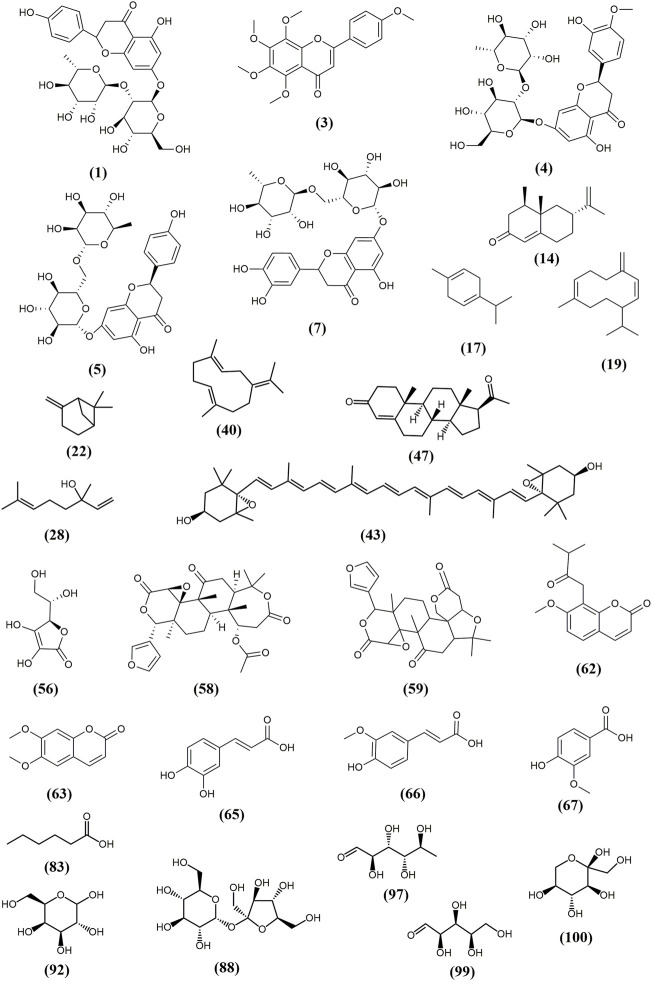
Selected structures of chemical constituents isolated from CACH.

Up to now, the chemical constituents of CACH have been widely investigated, more than 102 major compounds have been isolated and identified from it (selected into Figure 3). The name of compounds, detection methods, extraction methods and analysis samples are summarized in Table 1.

**TABLE 1:** Chemical constituents isolated from the different parts of CACH.

Name	[CAS]	Detection/extraction mode	Analysis parts of sample	References
Phenols and their derivatives				
Total phenols		HPLC; UV-Vis/Ultrasonic Treatment	Juice	Zhou et al. (2021)
		HS-SPME; GC-MS; HPLC/Pectin hydrolysates	Pulp	Lu et al. (2018)
		HPLC; LC-MS/Ethanol Extraction	Flavedo; Membrane; Juice	Hu et al. (2020)
Total flavonoids		UV-Vis/Methanol Extraction	Peel	Shu et al. (2017)
		HPLC; UV-Vis/Ultrasonic Treatment	Juice	Zhou et al. (2021)
		HS-SPME; GC-MS; HPLC/Pectin hydrolysates	Pulp	Lu et al. (2018)
Volatile compounds		HS-SPME; GC-MS; HPLC/Pectin hydrolysates	Pulp	Lu et al. (2018)
Naringin **(1)**	[10236–47–2]	HPLC/Methanol Extraction	Peel	Fang et al. (2018)
		HPLC; TLC/Ethanol Extraction	Dry peel	Hu et al. (2020)
		HPLC; LC-MS/Ethanol Extraction	Flavedo; Membrane; Juice	Zhang et al. (2012)
		HPLC-PDA/Methanol Extraction	Peel	Xu et al. (2007)
Hesperidin **(2)**	[520–26–3]	HPLC; TLC/Ethanol Extraction	Dry peel	Hu et al. (2020)
		HPLC-PDA/Methanol Extraction	Peel	Xu et al. (2007)
Tangeretin **(3)**	[481–53–8]	HPLC; TLC/Ethanol Extraction	Dry peel	Hu et al. (2020)
Neohesperidin **(4)**	[13241–33–3]	HPLC; UV-Vis/Ultrasonic Treatment	Juice	Zhou et al. (2021)
		HPLC; LC-MS/Ethanol Extraction	Flavedo; Membrane; Juice	Zhang et al. (2012)
		HPLC-PDA/Methanol Extraction	Peel	Xu et al. (2007)
Narirutin **(5)**	[14259–46–2]	HPLC/Methanol Extraction	Peel	Fang et al. (2018)
		HPLC; UV-Vis/Ultrasonic Treatment	Juice	Zhou et al. (2021)
		HPLC-PDA/Methanol Extraction	Peel	Xu et al. (2007)
Nobiletin **(6)**	[478–01–3]	HPLC; TLC/Ethanol Extraction	Dry peel	Hu et al. (2020)
Eriocitrin **(7)**	[13463–28–0]	HPLC/Methanol Extraction	QZQ	Feng et al. (2020)
		HPLC; UV-Vis/Ultrasonic Treatment	Juice	Zhou et al. (2021)
Luteolin **(8)**	[491–70–3]	HPLC/Methanol Extraction	QZQ	Feng et al. (2020)
Naringenin **(9)**	[67604–48–2]	HPLC/Methanol Extraction	QZQ	Feng et al. (2020)
		HPLC; UV-Vis/Ultrasonic Treatment	Juice	Zhou et al. (2021)
Neoeriocitrin **(10)**	[13241–32–2]	HPLC; UV-Vis/Ultrasonic Treatment	Juice	Zhou et al. (2021)
3,5,6,7,8,3',4'-Heptamethoxyflavone **(11)**	[1178–24–1]	HPLC; TLC/Ethanol Extraction	Dry peel	Hu et al. (2020)
(-)-Catechin Hydrate **(12)**	[18829–70–4]	HPLC/Water Extraction	Dry peel	Xu et al. (2008)
2,4-di-tert-butylphenol **(13)**	[96–76–4]	GC-MS; HPLC/Water and methanol extraction	Peel	Zhang et al. (2019)
Terpenes and carotenoids				
Total carotenoids		HPLC/Chloroform extraction	Peel	Luan et al. (2020)
Colorless total carotenoids		HPLC/Chloroform extraction	Peel	Luan et al. (2020)
Nootkatone **(14)**	[4674–50–4]	HPLC; TLC/Ethanol Extraction	Dry peel	Hu et al. (2020)
		GC-MS; HPLC/Water and methanol extraction	Peel; Juice	Zhang et al. (2019)
β-Myrcene **(15)**	[123–35–3]	GC-MS; HPLC/Water and methanol extraction	Peel; Juice	Zhang et al. (2019)
Limonene **(16)**	[138–86–3]	HS-SPME; GC-MS; HPLC/Pectin hydrolysates	Pulp	Lu et al. (2018)
		GC-MS; HPLC/Water and methanol extraction	Peel; Juice	Zhang et al. (2019)
γ-Terpinene **(17)**	[99–85–4]	GC-MS; HPLC/Water and methanol extraction	Peel; Juice	Zhang et al. (2019)
γ- Elemene **(18)**	[33880–83–0]	GC-MS; HPLC/Water and methanol extraction	Peel	Zhang et al. (2019)
Germacrene D **(19)**	[37839–63–7]	GC-MS; HPLC/Water and methanol extraction	Peel; Juice	Zhang et al. (2019)
Caryophyllene **(20)**	[87–44–5]	GC-MS; HPLC/Water and methanol extraction	Peel	Zhang et al. (2019)
α-Pinene **(21)**	[80–56–8]	GC-MS; HPLC/Water and methanol extraction	Peel	Zhang et al. (2019)
β-Pinene **(22)**	[127–91–3]	GC-MS; HPLC/Water and methanol extraction	Peel	Zhang et al. (2019)
α-Thujene **(23)**	[2867–05–2]	GC-MS; HPLC/Water and methanol extraction	Peel	Zhang et al. (2019)
Sabinene **(24)**	[3387–41–5]	GC-MS; HPLC/Water and methanol extraction	Peel	Zhang et al. (2019)
α-Phellandrene **(25)**	[99–83–2]	GC-MS; HPLC/Water and methanol extraction	Peel	Zhang et al. (2019)
β-cis-Ocimene **(26)**	[3338–55–4]	GC-MS; HPLC/Water and methanol extraction	Peel	Zhang et al. (2019)
α-Terpinene **(27)**	[99–86–5]	GC-MS; HPLC/Water and methanol extraction	Peel	Zhang et al. (2019)
Linalool **(28)**	[78–70–6]	GC-MS; HPLC/Water and methanol extraction	Peel; Juice	Zhang et al. (2019)
α-Terpineol **(29)**	[10482–56–1]	HS-SPME; GC-MS; HPLC/Pectin hydrolysates	Pulp	Lu et al. (2018)
		GC-MS; HPLC/Water and methanol extraction	Peel	Zhang et al. (2019)
Citronellal **(30)**	[106–23–0]	GC-MS; HPLC/Water and methanol extraction	Peel	Zhang et al. (2019)
Methyl 2-methyloctanoate **(31)**	[2177–86–8]	GC-MS; HPLC/Water and methanol extraction	Peel; Juice	Zhang et al. (2019)
Lavandulyl acetate **(32)**	[25905–14–0]	GC-MS; HPLC/Water and methanol extraction	Peel	Zhang et al. (2019)
Copaene **(33)**	[3856–25–5]	GC-MS; HPLC/Water and methanol extraction	Peel; Juice	Zhang et al. (2019)
Cubebene **(34)**	[13744–15–5]	GC-MS; HPLC/Water and methanol extraction	Peel	Zhang et al. (2019)
β-Elemene **(35)**	[515–13–9]	GC-MS; HPLC/Water and methanol extraction	Peel; juice	Zhang et al. (2019)
(E)-β-Famesene **(36)**	[18794–84–8]	GC-MS; HPLC/Water and methanol extraction	Peel	Zhang et al. (2019)
δ-Cadinene **(37)**	[29350–73–0]	GC-MS; HPLC/Water and methanol extraction	Peel	Zhang et al. (2019)
δ-EIemene **(38)**	[20307–84–0]	GC-MS; HPLC/Water and methanol extraction	Peel	Zhang et al. (2019)
Citronellol acetate **(39)**	[150–84–5]	GC-MS; HPLC/Water and methanol extraction	Peel	Zhang et al. (2019)
Germacrene B **(40)**	[15423–57–1]	GC-MS; HPLC/Water and methanol extraction	Juice	Zhang et al. (2019)
Methyl nonanoate **(41)**	[1731–84–6]	GC-MS; HPLC/Water and methanol extraction	Juice	Zhang et al. (2019)
Phytofluene **(42)**	[540–05–6]	GC-MS; HPLC/Water and methanol extraction	Peel; juice	Zhang et al. (2019)
		HPLC/Chloroform extraction	Peel	Luan et al. (2020)
Violaxanthin **(43)**	[126–29–4]	GC-MS; HPLC/Water and methanol extraction	Peel; juice	Zhang et al. (2019)
		HPLC/Chloroform extraction	Peel	Luan et al. (2020)
Luteoxanthin **(44)**	[101627–33–2]	GC-MS; HPLC/Water and methanol extraction	Peel; juice	Zhang et al. (2019)
Phytoene **(45)**	[540–04–5]	GC-MS; HPLC/Water and methanol extraction	Pulp	Zhang et al. (2019)
		HPLC/Chloroform extraction	Peel	Luan et al. (2020)
β-Carotene **(46)**	[7235–40–7]	HPLC/Chloroform extraction	Peel	Luan et al. (2020)
		GC-MS; HPLC/Water and methanol extraction	Peel; juice	Zhang et al. (2019)
Lutein **(47)**	[57–83–0]	HPLC/Chloroform extraction	Peel	Luan et al. (2020)
		GC-MS; HPLC/Water and methanol extraction	Peel; juice	Zhang et al. (2019)
Zeaxanthin **(48)**	[144–68–3]	HPLC/Chloroform extraction	Peel	Luan et al. (2020)
		GC-MS; HPLC/Water and methanol extraction	Peel; juice	Zhang et al. (2019)
β-Sitosterol **(49)**	[83–46–5]	HPLC; TLC/Ethanol Extraction	Dry peel	Hu et al. (2020)
β-sitosterol-D-glucoside **(50)**	[474–58–8]	HPLC; TLC/Ethanol Extraction	Dry peel	Hu et al. (2020)
β-Cryptoxanthin **(51)**	[472–70–8]	HPLC/Chloroform extraction	Peel	Luan et al. (2020)
		GC-MS; HPLC/Water and methanol extraction	Peel; juice	Zhang et al. (2019)
β-Citraurin **(52)**	[650–69–1]	HPLC/Chloroform extraction	Peel	Luan et al. (2020)
9-cis-Violaxanthin **(53)**	[26927–07–1]	HPLC/Chloroform extraction	Peel	Luan et al. (2020)
ζ-Carotene **(54)**	[72746–33–9]	HPLC/Chloroform extraction	Peel	Luan et al. (2020)
α-Carotene **(55)**	[7488–99–5]	HPLC/Chloroform extraction	Peel	Luan et al. (2020)
Vitamin C **(56)**	[50–81–7]	HS-SPME; GC-MS; HPLC/Pectin hydrolysates	Pulp	Lu et al. (2018)
Limonins and Coumarins				
6',7'-Dihydroxybergamottin **(57)**	[145414–76–2]	IR; MS; TLC/Ethanol Extraction	Peel	Feng et al. (2020)
Nomilin **(58)**	[1063–77–0]	HPLC/Methanol Extraction	QZQ	Feng et al. (2020)
Limonin **(59)**	[1180–71–8]	HPLC; TLC/Ethanol Extraction	Dry peel	Hu et al. (2020)
(-)-8-[(S)-2,3-Dihydroxy-3-methylbutyl]-7-methoxy-2H-1-benzopyran-2-one **(60)**	[5875–49–0]	HPLC/Methanol Extraction	QZQ	Feng et al. (2020)
Auraptene **(61)**	[495–02–3]	HPLC/Methanol Extraction	QZQ	Feng et al. (2020)
Meranzin **(62)**	[23971–42–8]	HPLC/Methanol Extraction	QZQ	Feng et al. (2020)
Scoparone **(63)**	[120–08–1]	HPLC; TLC/Ethanol Extraction	Dry peel	Hu et al. (2020)
Organic acids				
Total cinnamics and benzoics		HPLC-PDA/Methanol Extraction	Peel	Xu et al. (2007)
p-Coumaric **(64)**	[501–98–4]	HPLC-PDA/Methanol Extraction	Peel	Xu et al. (2007)
Caffeic acid **(65)**	[331–39–5]	HPLC-PDA/Methanol Extraction	Peel	Xu et al. (2007)
Ferulic Acid **(66)**	[1135–24–6]	HPLC-PDA/Methanol Extraction	Peel	Xu et al. (2007)
Vanillic acid **(67)**	[121–34–6]	HPLC-PDA/Methanol Extraction	Peel	Xu et al. (2007)
p-Hydroxybenzoic **(68)**	[99–96–7]	HPLC-PDA/Methanol Extraction	Peel	Xu et al. (2007)
Chlorogenic **(69)**	[327–97–9]	HPLC-PDA/Methanol Extraction	Peel	Xu et al. (2007)
Sinapic **(70)**	[530–59–6]	HPLC-PDA/Methanol Extraction	Peel	Xu et al. (2007)
Carbamic acid **(71)**	[59812–12–3]	GC-MS; HPLC/Water and methanol extraction	Peel	Zhang et al. (2019)
Cyclohexaneacetic acid **(72)**	[5292–21–7]	GC-MS; HPLC/Water and methanol extraction	Peel	Zhang et al. (2019)
Malic acid **(73)**	[6915–15–7]	GC-MS; HPLC/Water and methanol extraction	Peel; Juice	Zhang et al. (2019)
Quinic acid **(74)**	[77–95–2]	GC-MS; HPLC/Water and methanol extraction	Peel; Pulp	Zhang et al. (2019)
Quininic acid **(75)**	[86–68–0]	GC-MS; HPLC/Water and methanol extraction	Juice	Zhang et al. (2019)
Shikimic acid **(76)**	[138–59–0]	GC-MS; HPLC/Water and methanol extraction	Peel	Zhang et al. (2019)
2-Ketoglutaric acid **(77)**	[328–50–7]	GC-MS; HPLC/Water and methanol extraction	Peel	Zhang et al. (2019)
4-Aminobutanoic acid **(78)**	[56–12–2]	GC-MS; HPLC/Water and methanol extraction	Peel	Zhang et al. (2019)
Palmitic acid **(79)**	[57–10–3]	GC-MS; HPLC/Water and methanol extraction	Peel	Zhang et al. (2019)
Oxalic acid **(80)**	[144–62–7]	GC-MS; HPLC/Water and methanol extraction	Juice	Zhang et al. (2019)
Citric acid **(81)**	[77–92–9]	GC-MS; HPLC/Water and methanol extraction	Juice	Zhang et al. (2019)
Acetic acid **(82)**	[64–19–7]	HS-SPME; GC-MS; HPLC/Pectin hydrolysates	Pulp	Lu et al. (2018)
Hexanoic acid **(83)**	[142–62–1]	HS-SPME; GC-MS; HPLC/Pectin hydrolysates	Pulp	Lu et al. (2018)
Nonanoic acid **(84)**	[112–05–0]	HS-SPME; GC-MS; HPLC/Pectin hydrolysates	Pulp	Lu et al. (2018)
n-Decanoic acid **(85)**	[334–48–5]	HS-SPME; GC-MS; HPLC/Pectin hydrolysates	Pulp	Lu et al. (2018)
Dodecanoic acid **(86)**	[143–07–7]	HS-SPME; GC-MS; HPLC/Pectin hydrolysates	Pulp	Lu et al. (2018)
Sugars				
Reducing sugar **(87)**		HS-SPME; GC-MS; HPLC/Pectin hydrolysates	Pulp	Lu et al. (2018)
Sucrose **(88)**	[57–50–1]	GC-MS; HPLC/Water and methanol extraction	Jucie; Peel	Zhang et al. (2019)
Arabinose **(89)**	[147–81–9]	GC-MS; HPLC/Water and methanol extraction	Jucie; Peel	Zhang et al. (2019)
Turanose **(90)**	[547–25–1]	GC-MS; HPLC/Water and methanol extraction	Jucie; Peel	Zhang et al. (2019)
Mannose **(91)**	[530–26–7]	GC-MS; HPLC/Water and methanol extraction	Jucie; Peel	Zhang et al. (2019)
Galactose **(92)**	[10257–28–0]	GC-MS; HPLC/Water and methanol extraction	Peel	Zhang et al. (2019)
Fucose **(93)**	[7724–73–4]	GC-MS; HPLC/Water and methanol extraction	Peel	Zhang et al. (2019)
Fructose **(94)**	[57–48–7]	GC-MS; HPLC/Water and methanol extraction	Jucie; Peel	Zhang et al. (2019)
d-Psicose **(95)**	[551–68–8]	GC-MS; HPLC/Water and methanol extraction	Peel	Zhang et al. (2019)
Myo-Inositol **(96)**	[87–89–8]	GC-MS; HPLC/Water and methanol extraction	Jucie; Peel	Zhang et al. (2019)
Rhamnose **(97)**	[3615–41–6]	GC-MS; HPLC/Water and methanol extraction	Jucie	Zhang et al. (2019)
D-glucose **(98)**	[128705–73–7]	GC-MS; HPLC/Water and methanol extraction	Jucie	Zhang et al. (2019)
Xylose **(99)**	[41247–05–6]	GC-MS; HPLC/Water and methanol extraction	Jucie; Peel	Zhang et al. (2019)
Sorbose **(100)**	[87–79–6]	GC-MS; HPLC/Water and methanol extraction	Jucie	Zhang et al. (2019)
Others				
Trace Elements **(101)**		AAS/Water Extraction	Dry peel	Xu et al. (2008)
Protein**(102)**		HS-SPME; GC-MS; HPLC/Pectin hydrolysates	Fermented wine	Lu et al. (2018)

### 4.1 Phenols and their derivatives

**FIGURE 4:**
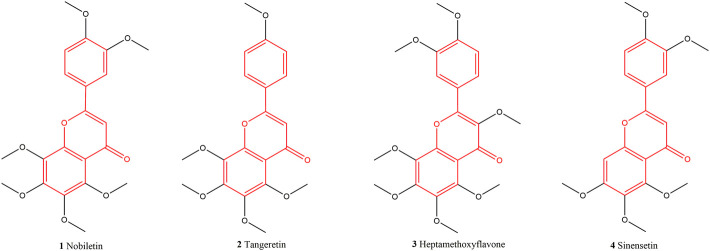
Four kinds of PMFs isolated from citrus.

Phenolic compounds in citrus peel mainly include flavonoids and phenolic acids, such as naringin (1), hesperidin (2), neohesperidin (4), tangeretin (3), nobiletin (6), etc., (Singh et al., 2020). Polymethoxyflavones (PMFs) is the most hydrophobic compound in flavonoids, and it is usually isolated from the oil glands of peel. Li et al. (2019) isolated four PMFs from citrus (Figure 4). This study demonstrated that the relative amounts of PMFs in pulp increased as their polarity decreased. Ballistreri (2019) believed that pulp fractions also contained phenolic compounds, but the amount was relatively low. The flavonoids in pulp mainly exist in the form of glycosides, while Citrus peel is abundant in the less polar flavanone as well as flavonoid aglycones and PMFs (11).

To date, 13 major phenolic compounds and their glycosides have been isolated and identified from CACH (the other compounds were not included in this statistics due to their low activity or yield) (No. 1–13, Table 1). Modern studies have indicated flavonoids are representative and dominant phenolic compounds isolated from CACH. Most of the flavonoids in it were in the form of flavanone glycosides, such as naringin (1), neohesperidin (4), narirutin (5), eriocitrin (7), neoeriocitrin (10), PMFs (11), and tangeretin (3) etc., (Hu et al., 2020; Zhou et al., 2021).

### 4.2 Terpenes and carotenoids

Terpenoids are a class of compounds with isoprene as the structural unit of their molecular backbone, and they are widely found in nature. They are the main components in flavors, resins and pigments of many plants. Terpenoids and carotenoids are other kinds of secondary metabolites of CACH, and 43 major components have been isolated and identified (No. 14–56, Table 1).

Nootkatone (14) is one of the characteristic aroma components in the peel of CACH. Zhang et al. (2019) analyzed the volatile components and carotenoids in the peel and juice of CACH. They identified 36 compounds including monoterpenes and sesquiterpenes, such as β-Myrcene (15), γ-elemene, Sabinene (24) and α-terpinene (27),etc., At the same time, they found phytoene of eight carotenoids was detected only in juice. Besides tetraterpenoid carotenoids, monoterpenoid, sesquiterpenoid volatiles and triterpenoid bitter compounds were also important secondary metabolites of citrus fruit (Liu et al., 2015).

### 4.3 Limonins and coumarins

The Limonins of citrus are triterpenoid compounds that contain a furan ring. They are secondary metabolites of some high-oxygen terpenoids. As one of the bitter substances in most citrus, it exists in seeds, pulp and peel, and the order of its content in different parts is seed > peel > pulp (Gualdani et al., 2016). There are more than 50 limonin compounds isolated and identified from citrus, including nomilin (58), limonin (59), nomilin acid and their glycosides (Lam et al., 1989). At present, the biogenetic relationships of 15 limonin precursors and limonin homologies in citrus have been confirmed (Lakshmi and Gupta, 2008; Heasley, 2011).

Coumarins are some compounds with the basic structure of 4-hydroxycoumarin. Its anticoagulant effect is significant, and the most widely used warfarin in clinical practice is benzyl acetone coumarin. At present, twelve coumarins and furanocoumarins were isolated from the fruit juice, pulp and flesh of citrus fruits (Li et al., 2019) (Table 2). Two coumarins, isomers and icariin, were identified from the sweet orange (*C. sinensis*). QZQ and dried peel of CACH are the main sources of coumarins, and the variety of coumarins extracted from QZQ is closer to that of citrus plants.

**TABLE 2:** Coumarins and furanocoumarins isolated from citrus.

Coumarins	Name	R1	R2	R3	R4
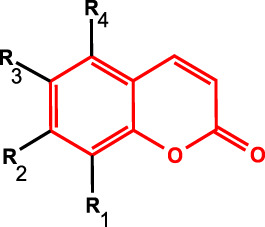	Isomeranzin	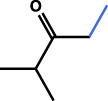	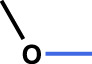	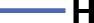	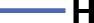
	Scopoletin	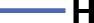	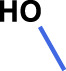	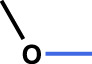	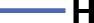
	Citropten	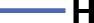	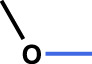	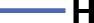	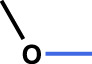
	Osthole	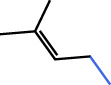	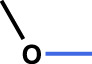	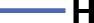	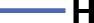
	Auraptene	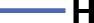	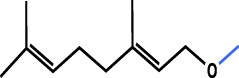	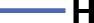	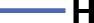
	Meranzin	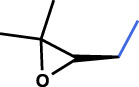	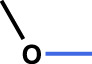	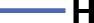	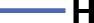

Furanocoumarins	**\**	**R**			
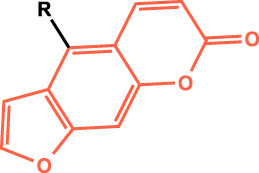	Bergaptol	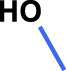			
	Oxypeucedanin	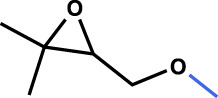			
	Bergapten	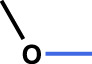			
	Epoxybergamottin	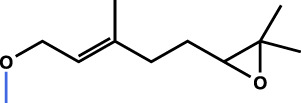			
	6′,7′-dihydroxy bergamottin	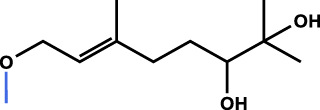			
	Bergamottin	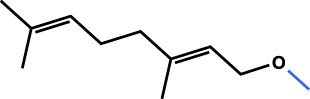			

### 4.4 Other compounds

In addition to the above components, there are various known complex components in CACH, such as organic acids (Lu et al., 2018) (No. 64–86, Table 1), amino acids (Zheng et al., 2016), sugars (Zhang et al., 2019), and inorganic elements (101) (Xu et al., 2008). Some studies have been reported on the content and types of them. For example, it was found that CACH contains 16 kinds of amino acids, including eight kinds of essential amino acids, and the content of ASP is the highest (Zheng et al., 2016). Zhang et al. (2019) conducted the type identification and content detection of sugars in CACH. These studies provided an extremely important theoretical basis for the industrial development of CACH, and promoted the further development of its medical and food industry.

## 5 Pharmacological effects

**FIGURE 5:**
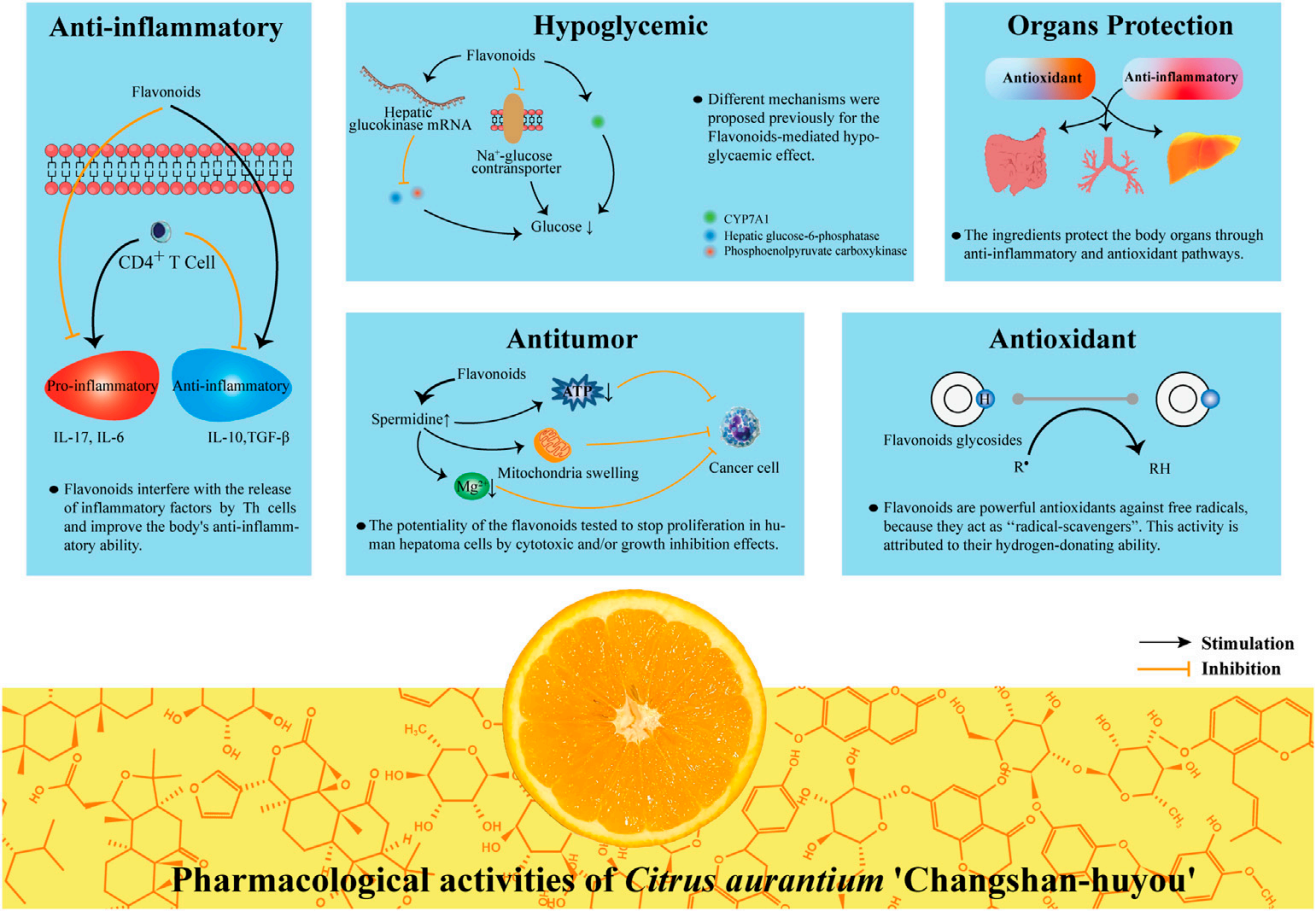
The pharmacological activities of CACH.

The production of CACH is large and the storage is abundant. The ethnomedical applications of CACH has attracted the attention of the scientific community. It stimulates the trend of in-depth research on various pharmacological mechanisms of CACH. Currently known extracts and isolated compounds have various pharmacological effects, such as anti-inflammatory, antioxidant, antitumor, hypolipidemic and protection on organs. The specific pharmacological activities are shown in Table 3 and summarized as follows (Figure 5).

**TABLE 3:** Pharmacological effects of CACH.

Crude Drug/compounds	Model method	Dose range/concentration	Results	References
Antioxidant Activity				
Flavonoids in QZQ	Female BALB/C mice infected with RSV	20–80 mg/kg·d^−1^ i.g	The mechanism of antioxidant effect may be related to NF-κB signaling pathway	Liu et al. (2021)
TFCH	HFD-induced male C57BL/6 mice; free fatty acid-induced LX-2 cells	Cell:25–200 mg/ml	The antioxidant effect increased with increasing dose	Shi et al. (2020)
		Mice:25–100 mg/kg·d^−1^ i.g		
PTFC	HFD-induced male LVG Syrian golden hamsters	25–100 mg/kg·d^−1^ i.g	SOD and MDA tests proved that PTFC could reduce the level of oxidative stress	Ling et al. (2020)
Anti-inflammatory Activity				
PTFC	LVG male golden hamster	25–100 mg/kg·d^−1^ i.g	PTFC could down-regulate the serum inflammatory factors, and the 100 mg/kg·d^−1^ group has the best effect	Ye et al. (2020)
Components of Dry peel	lipopolysaccharide-induced RAW264.7 cell	20 μM	Various derivatives of nootkatone, scoparone and limonin showed inhibitory effect on TNF-α	Hu et al. (2020)
TFCH	OVA-induced mice model	25–100 mg/kg·d^−1^ i.g	TFCH suppresses OVA-induced inflammatory cells recruitment	Wang et al. (2021)
TFCH	HFD-induced Male SD rats	25–100 mg/kg·d^−1^ i.g	The anti-inflammatory effect increases with dose	Jiang et al. (2019)
PTFC	HFD-induced male LVG Syrian golden hamsters	25–100 mg/kg·d^−1^ i.g	PTFC reduced inflammatory damage by reducing levels of TNF-α and IL-6 in serum and liver cells	Ling et al. (2020)
Hepatoprotective Activity				
PTFC	HFD indu ced male C57BL/6 mice	25–100 mg/kg·d^−1^ i.g	PTFC could delay the progression of NAFLD by inhibiting NLRP3. 100 mg/kg·d^−1^ is significant	Yu et al. (2019)
PTFC	HFD induced male C57BL/6 mice	25–100 mg/kg·d^−1^ i.g	PTFC improved liver function by improving mitochondrial dysfunction	Chen et al. (2014)
TFCH	OVA-induced mice	25–100 mg/kg·d^−1^ i.g	TFCH inhibited airway inflammation and remodeling in allergic asthma	Wang et al. (2021)
PTFC	HFD-induced C57BL/6 J mice	50 mg/kg·d^−1^ i.g	PTFC could improve NASH via the gut microbiota and bile acid metabolism	He et al. (2021)
Hypoglycemic activity				
PTFC	HFD-induced male SD rats	50–200 mg/kg·d^−1^ i.g	PTFC could regulate lipid metabolism by improving antioxidant capacity of the mice	Yang et al. (2017)
Purification of naringin and neohesperidin from CACH	HepG2 cells; Glucose consumption assay	0.5–25 μg/ml	Cells treated with naringin and neohesperidin showed increased consumption of glucose	Zhang et al. (2012)
PTFC	LVG male golden hamster	25–100 mg/kg·d^−1^ i.g	PTFC could improve hepatic steatosis of hyperlipidemia golden hamsters, and 100 mg/kg·d^−1^ group has the best efficacy	Ye et al. (2020)
PTFC	HFD-induced male LVG Syrian golden hamsters	25–100 mg/kg·d^−1^ i.g	PTFC significantly increased aspartate aminotransferase and alkaline phosphatase levels related to liver function of golden hamsters	Ling et al. (2020)
Respiratory protection				
TFCH	OVA-induced mice	25–100 mg/kg·d^−1^ i.g	TFCH suppresses OVA-induced inflammatory cells recruitment	Wang et al. (2021)
Total flavonoids of CACH	Female BALB/C mice infected with RSV	20–80 mg/kg·d^−1^ i.g	With the increase of concentration, the effect of improving asthma increased	Liu et al. (2021)
Intestinal adjustment				
Fermented CACH juice	HFD-induced C57BL/6 J mice; expressed genes in GO enrichment	10 mg/kg·d^−1^	The juice ameliorated the gut dysbiosis caused by obesity. When receiving fermented CACH juice treatment, a dramatic decrease in Firmicutes/Bacteroidetes occurred	Yan et al. (2020)
PTFC	HFD-induced C57BL/6 J mice	50 mg/kg·d−1 i.g	PTFC treatment increased the phylogenetic diversity of the HFDinduced microbiota dysbiosis	He et al. (2021)
PTFC	Diclofenac-Induced male SD rats; Diclofenac sodium-Induced IEC-6	Cell: 1 mg/ml	PTFC treatment alleviates NSAIDS induced intestinal injury and protects the intestinal mucosal barrier in rats by upregulating autophagy	Chen et al. (2021)
		Rat: 100 mg/kg/d i.g		

### 5.1 Antioxidant activity

Flavonoids are powerful antioxidants against free radicals, because they act as “radical-scavengers”. This activity is attributed to their hydrogen-donating ability (Burda and Oleszek, 2001). Majo et al. (2005) found that the phenolic groups of flavonoids served as a source of a readily available “H” atoms such that the subsequent radicals produced could be delocalized over the flavonoid structure.

Several studies have shown that hesperidin has the activity of enhancing the antioxidant defense ability of cells (Martínez et al., 2009; Elavarasan et al., 2012). According to the research of (Shi et al., 2020), total flavonoids of QZQ (TFCH) had obvious antioxidant effects on the nonalcoholic steatohepatitis (NASH) model of high fat diet (HFD)-induced C57BL/6 mice and free fatty acid-induced cells. Specifically, the authors made a botanical identification of QZQ (Voucher: JJ, 101011, ZM). Then the processed QZQ was repeatedly extracted with Ca(OH)_2_ at 100°C, and the filtrate was enriched with HPD-300 macroporous resin to obtain 3.83 mg/ml TFCH. By using rutin equivalent, the purity of TFCH determined by HPLC was 76.22%. Finally, they calculated the contents of narirutin (5), naringin (1) and neohesperidin (4), which contained 12.08 ± 0.12 mg/g, 243.86 ± 2.67 mg/g, and 136.02 ± 4.55 mg/g, respectively. Interestingly, total antioxidant capacity, superoxide dismutase (SOD), glutathione (GSH) peroxidase activities of cells and mice were increased after treatment with TFCH in the dose range of 25–200 mg/ml and 25–100 mg/kg, meanwhile the expression levels of malondialdehyde (MDA) and 8-iso-PGF2α in serum were opposite. The results were also confirmed in positive drug group (Vitamin E group) of the research, and the TFCH high-dose group (100 mg/kg) have better regulation than Vitamin E group (100 mg/kg). In addition, the expression of antioxidant enzymes (HO-1, glutathione S-transferases, NQO1, γ-GCS) in cells and mice liver were increased with the increase of TFCH concentration. These results suggest that total flavonoids may be the material basis of antioxidant activity of CACH. However, the specific mechanism of action need to be further confirmed.

### 5.2 Anti-inflammatory activity

**FIGURE 6:**
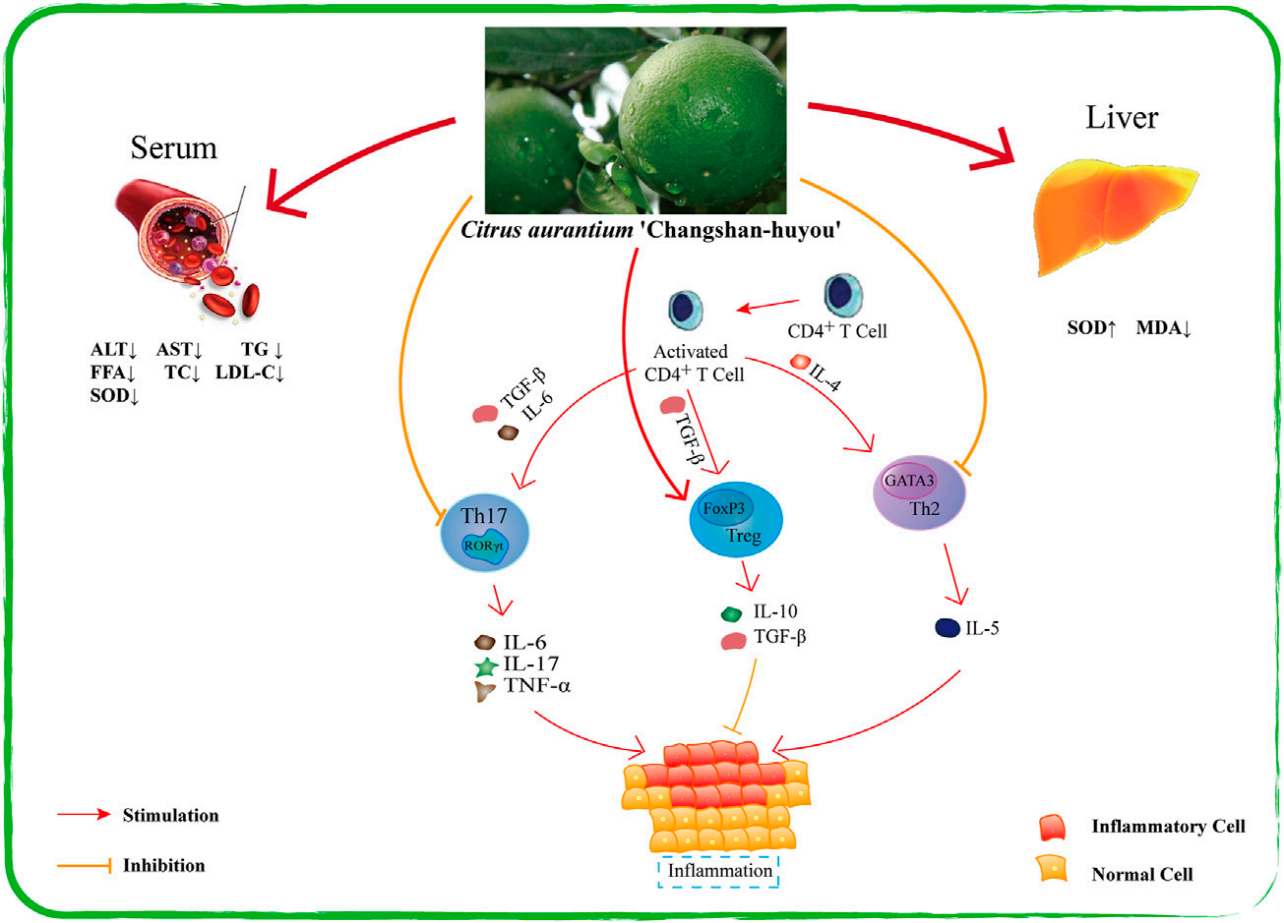
Summary of anti-inflammatory mechanism of CACH.

CACH could be used in the treatment of chronic gastritis and peptic ulcer, these diseases are mainly related to the overexpression of inflammatory factors. Jiang et al. (2019) carried out the *in vivo* anti-inflammatory effect of TFCH. The specific method was to induce male SD rats with HFD, and then used TFCH treated rats. The results showed that TFCH at 50–100 mg kg^−1^ significantly decreased the expression of TNF-α, IL-6, IL-1β and other inflammatory factors. NF-κB/MAPK was proposed in the study, which is a potential key pathway for TFCH to express anti-inflammatory effects. Wang et al. (2021) used TFCH to intervene in BALB/c mouse model of allergic asthma induced by ovalbumin (OVA), confirmed and described the anti-inflammatory effect and mechanism of CACH. More specifically, the anti-inflammatory factors IL-4, IL-13 were increased in the lung tissues of mice after treating with TFCH. Meanwhile authors counted the number of inflammatory cells in bronchoalveolar lavage fluid (BALF) via Swiss-Giemsa staining. It found that TFCH significantly altered the numbers of total leukocytes, eosinophils, monocytes, neutrophils, and lymphocytes in BALF in a dose-dependent manner, and improved the inflammatory microenvironment of bronchoalveolar cells. Based on the above findings, this paper summarizes the current studies on the anti-inflammatory mechanism of CACH, as shown in Figure 6.

### 5.3 Hypoglycemic activity

In recent years, the preventive and therapeutic effects of CACH on diabetes and hyperlipidemia received extensive attention and research. Although its mechanism has not been fully elucidated, its hypoglycemic effect may be achieved by increasing glucose consumption, regulating intestinal flora, and improving lipid metabolism.

Early research showed that the intervention of naringin and neohesperidin increased intracellular glucose consumption, a process associated with increased phosphorylation of AMP-activated protein kinase (Zhang et al., 2012). According to the literature of (Ling et al., 2020), the authors extracted PTFC from CACH (the same method as TFCH), and fed male LVG Syrian golden hamsters with HFD to establish hyperlipidemia model. It was found that PTFC (25, 50, 100 mg/kg) interfered with the protein expression level of PPAR-α/PPAR-γ and restored the content of cholesterol metabolic enzymes cholesterol 7a-hydroxylase (CYP7A1). After comparing the positive drug control group (fenofibrate, 100 mg/kg), they concluded that PTFC could improve lipid metabolism, reduce pathological damage caused by hyperlipidemia, and reduce blood glucose level.

### 5.4 Antitumor activity

The proliferation and metastasis of cancer cells are mediated by many factors. Modern researchs have made the effort on tumor microenvironment, pathogenesis and biomarkers for a long time, but the development of highly specific antitumor drugs is still one of the bottlenecks in this field (Mashouri et al., 2019). The use of diet to treat cancer has been a new research direction in recent years. From the perspective of regulating the cancer metabolic microenvironment, the method of antitumor is to find out the active molecules of food, which can specifically target metabolic pathway. As mentioned above, CACH contains abundant flavonoids, including neohesperidin (4) and PMFs (11). Interestingly, Interestingly, early studies have shown that neohesperidin has neuroprotective activity (Hwang and Yen, 2008; Martínez et al., 2009) and antiproliferative effect on human hepatoma cells (Bellocco et al., 2009). Duan et al. (2017) isolated 5-hydroxy-6, 7, 8, 3 ', 4 '-pentamethoxyl flavonoids from citrus, and detected it had significant antiproliferative effect in tumor cell lines at 40 μM. According to the literatures in the database, it is found that the anti-tumor activity of CACH is related to intervening cell cycle and inhibiting proliferation. The targets and toxicological datas in this process still need to be further explored. More and more in-depth research on antitumor activity may be one of the future directions and trends.

### 5.5 Organs protection

As a fructus aurantii of Chinese herbals, QZQ has the characteristics of multi-target, multi-pathway, synergistic effect, non-toxicity, which are great value for the development of new drugs. Although it will change the types and content of active components after the TCM processing of CACH into QZQ, literatures shown that both of them have significant protection effect on organs, such as respiratory system protection, intestinal adjustment, hepatoprotective activity, etc. In these aspects, QZQ has direct or indirect regulatory effect on lung, stomach and other organs, which is not particularly different from the traditional effect of conventional fructus aurantii. The specific research contents are summarized as follows.

#### 5.5.1 Hepatoprotective activity

Previous studies have found CACH has protective effects on some liver disease models. The main disease models were nonalcoholic fatty liver disease (NAFLD) and liver fibrosis in these studies. The main pathological feature of NAFLD is diffuse hepatic fatty lesion, the disease spectrum includes simple fatty liver, steatohepatitis, and hepatic sclerosis, and some patients may even develop liver cancer (Yu et al., 2019). The inflammatory and oxidative stress’ microenvironment of liver is the main reasons for the development and rapid deterioration of NAFLD. Sufficient data indicate the prevention and treatment activities of CACH on liver diseases are mainly achieved through anti-inflammatory, antioxidant and intestinal microflora regulation.

**FIGURE 7:**
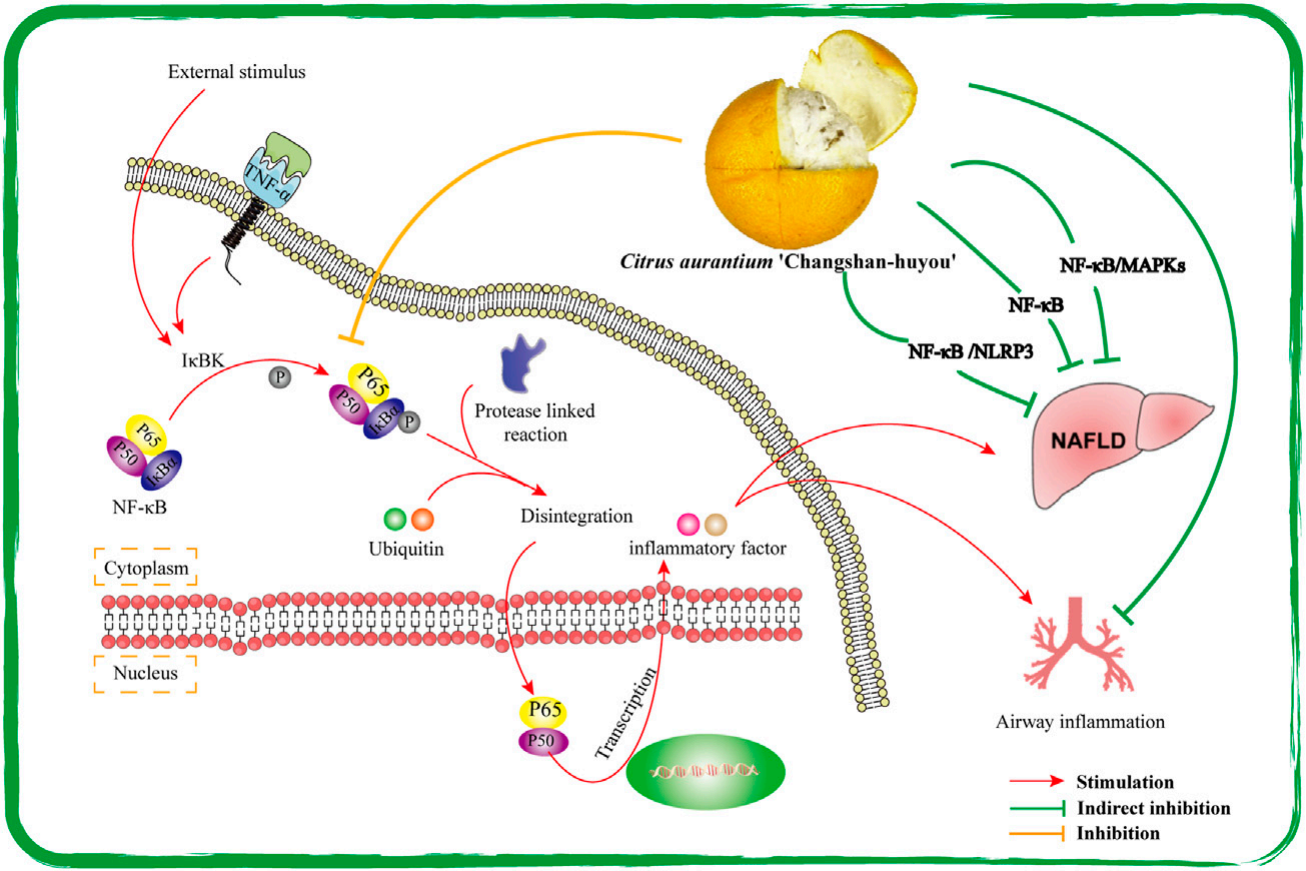
Protective mechanism of CACH on organs through NF-κB pathway.

For example, NF-κB, the key protein in the mechanisms of regulating liver inflammation in NAFLD, has been widely studied. TFCH was extracted and prepared by Jiang et al. (2019) and NAFLD model of male SD rats was constructed by HFD induction. TFCH improves the inflammatory environment of the liver by inhibiting the phosphorylation of IκBα to block the disintegration process of NF-κB, thereby inhibiting the synthesis and release of inflammatory factors. The results were also confirmed in positive drug group (Polyene phosphatidycholine capsule group, 196.3 mg/kg) of the research (Figure 7). In addition, Shi et al. (2020) found that antioxidant genes HO-1, NQO1, r-GCS, and GST of rats were upregulated after treating with TFCH. Unfortunately, the intermolecular regulatory role of these mechanisms has not been clearly elucidated.

#### 5.5.2 Respiratory system protection

Airway inflammation is the most common pathological feature of respiratory diseases. Allergic asthma is one of the typical diseases of respiratory system caused by chronic inflammation. It is triggered by some external factors, leading to infiltration of immune and inflammatory cells and accumulation in the airway. Wheezing, shortness of breath, chest tightness and bronchoconstriction are the diagnostic characteristics of it in clinic (Humbert et al., 2019). The flavonoids extract of CACH could alleviate local irritation by inhibiting the synthesis and release of inflammatory factors in airway smooth muscle. (Wang et al., 2021) found that TFCH showed anti-inflammatory activity and inhibited airway remodeling. It provides a potential therapeutic strategy for allergic asthma. Liu et al. (2021) verified the protective effect of TFCH on lung injury in asthmatic mice. They chose MAPK/Smad2 and 3 signaling pathways and found that TFCH could down-regulate IFN-γ and Th2 cytokines. Therefore, the deterioration of airway inflammation was inhibited, and airway pathology and hyperresponsiveness were improved. Relevant studies on the regulation of QZQ on respiratory system are important to the industrial development of CACH. They prove that the local folk prescriptions in Changshan are meaningful, and also provide scientific basis for the rational use of QZQ.

#### 5.5.3 Intestinal adjustment

Intestinal adverse reactions caused by drugs are very common and have been paid more and more attention by pharmaceutical industry. Reasonable intervention of intestinal microenvironment is of great significance to guide rational drug use in clinic. (Chen et al., 2014; Chen et al., 2021) studied the mechanism of small intestinal side reactions induced by non-steroidal antiinflammatory drugs (NSAIDs) *in vivo*, concluded that the integrity of intestinal barrier of the mice would be protected after treating with PTFC. Authors induced intestinal injury in SD rats via continuous irrigation stomach with diclofenac (7.5 mg/kg). Compared with the model group, it was found that the intestinal mucosa of rats in PTFC group (100 mg/kg) was only slightly congested and edematous. Meanwhile, administration of PTFC attenuated the decrease in intestinal tight junction protein expression and was associated with intestinal mucosal barrier repair in the NSAID-induced small intestine injury model. He et al. (2021) found that PTFC treatment increased the phylogenetic diversity of the HFD-induced microbiota dysbiosis. It could significantly increased the relative abundances of Bacteroidaceae and Christensenellaceae. Furthermore, PTFC reduced the content of toxic bile acids and increased the ratio of secondary to primary bile acids.

## 6 Discussion and conclusion

Rutaceae plants have a long medicinal history. They are widely used in many traditional Chinese medicine prescriptions and have been widely recognized in the clinical practice of TCM. Citrus fruits are grown in tropical, subtropical and temperate regions of the Earth. In recent years, they have become more and more popular in the World, and their rich bioactive substances have made a significant contribution to global human health.

Daily consumption of citrus fruits is one of the ideal dietary approaches to prevent diseases, which is related to the intervention of inflammatory production, antigen presentation, antioxidant defense mechanisms and intestinal microbiota. QZQ is the dry and immature fruit of CACH, a hybridization of *Citrus grandis* Osbeck and *C. sinensis* Osbeck. It has the function of promoting blood circulation and is used in TCM clinical treatment. Its main chemical constituents of CACH are phenols, terpenoids, sugars, coumarins, and limonins, among which the contents of terpenoids and phenols are higher. As a fructus aurantii of Chinese herbals, CACH shows a variety of significant pharmacological activities, such as anti-inflammatory, antioxidant, antitumor and hypolycemic activities, which provide a certain pharmacological basis for its clinical application. Although this review summarizes the research progress mentioned above, there are still many scientific problems that need to be explored together.

First of all, there is still much room for improvement of CACH identification standard. Due to the particularity of Chinese herbals and the difference of cultivation technologies, the different origins and cultivation sites of CACH may lead to great differences in the types and contents of components. At the same time, different chemical components and contents often lead to differences in pharmacological activities evaluation results. Based on these contradictory premises, the extracts used in some research didn’t mention their extraction methods and process conditions, and even some studies didn’t provide evidence for the identification of CACH or the description of the extraction site was not completely accurate. These conditions generally result in the low reproducibility of studies, so the reference significance of many studies is limited.

Secondly, the “multi-component” characteristics of CACH need more comprehensive and reliable theoretical support. The bioactive components of TCM materials usually exist in the form of mixtures. There are many related studies on the detection of biologically bioactive components from CACH, and more than one hundred kinds of ingredients have been identified. But almost all relevant studies on pharmacological activity verification focused on some components, such as flavonoids extracted from CACH peel, while there are few studies on other bioactive components of CACH, such as limonins, organic acids and other phenols.

Finally, the “multi-target, comprehensive intervention” advantages of CACH need further scientific and reasonable proof. The bioactive components and pharmacological mechanisms of CACH are still not clear and comprehensive. It is more common that researchers focus too much on the results. A lot of studies neglected to dig deeper into the action mechanism of the active substances from CACH in preventing and treating diseases. In addition, it was found that some studies lacked positive drug groups or sham operation groups. And some cells lines or animals used in some studies didn’t provide a complete and credible source basis. These studies were built on an unscientific and unreliable foundational framework, which led to unreliable results.

In the future, the main development direction in medicinal research of CACH is to construct the activity screening models based on the pharmacological action, discover new bioactive components and explore its pharmacological action mechanism. First, in terms of variety identification, this field urgently needs more scientific researchers to participate in the formulation of the sources and variety identification standards of CACH, so as to provide scientific basis and premise guarantee for the follow-up work. Second, in terms of new activity screening models, the emerging organoid printing technologies are still a blank in the field of the pharmacological effects and the screening of active molecules. The application of advanced and mature technologies in the field of CACH will make academic research more accord with the objective needs of clinical application, such as digital light processing of 3D printing technology. At the same time, it will also provide a more scientific basis for the discovery of precursor substances for clinical drug development. Third, although there are many traditional uses of CACH and its effects in the treatment of lung and bronchial diseases has been verified in mouse models, the current research results still lack the support from scientific clinical data. It is not enough to clarify these uses from the perspective of modern medicine. Therefore, it is necessary to obtain its toxicology and pharmacokinetics data, so as to provide a safe basis for clinical research and product development of CACH.

In conclusion, the review summarized the basic background, chemical composition, pharmacological activity, development bottleneck and future direction of CACH. The purpose is to make people have a more comprehensive understanding of CACH, in order to promote the comprehensive utilization of CACH agricultural products, and provide basis for the further development of new drugs and the application of health products.
